# Emerging Roles of Extracellular Vesicles in the Pathogenesis, Diagnosis, and Therapy of Periodontitis

**DOI:** 10.3390/biomedicines13102521

**Published:** 2025-10-16

**Authors:** Yiru Fu, Mengmeng Wang, Rui Teng, Ang Li

**Affiliations:** 1 Key Laboratory of Shaanxi Province for Craniofacial Precision Medicine Research, College of Stomatology, Xi’an Jiaotong University, Xi’an 710049, China; 2 Department of Orthodontics, College of Stomatology, Xi’an Jiaotong University, Xi’an 710049, China; 3 Department of Periodontology, College of Stomatology, Xi’an Jiaotong University, Xi’an 710049, China

**Keywords:** periodontitis, extracellular vesicles, pathogenesis, diagnosis, therapy

## Abstract

Periodontitis is a globally prevalent oral disease and is closely associated with various systemic diseases. Periodontitis arises from dynamic and complex interactions between polymicrobial communities and host immune responses. Extracellular vesicles (EVs) are circulating subcellular particles carrying multiple signaling molecules. EVs play a key role in intercellular communication, and hold promise for diagnostic and therapeutic purposes. Bacterial extracellular vesicles (BEVs), released from oral pathogens, have been implicated in delivering virulence factors to host cells. In contrast, host cell-derived EVs (CEVs), secreted by periodontal cells, contain molecular cargo that reflect disease status. Both BEVs and CEVs contribute to periodontitis progression by exacerbating inflammation and tissue destruction, and they may also influence related systemic diseases. Moreover, the molecular components of EVs derived from saliva and gingival crevicular fluid (GCF) show potential as diagnostic biomarkers for periodontitis. In addition, mesenchymal stem cell-derived EVs (MSC-EVs) exhibit therapeutic potential in periodontitis, and engineering approaches have been developed to enhance their therapeutic efficacy and accelerate clinical translation. This review summarizes recent advances in understanding the pathogenic, diagnostic, and therapeutic roles of EVs in periodontitis and discusses current challenges and future directions toward their clinical application.

## 1. Introduction

Periodontitis is a highly prevalent oral disease [1], affecting nearly 62% of the global population [2]. Recent data from the Global Burden of Disease Study 2021 reported that in 2021 there were approximately 951 million cases worldwide, with the highest prevalence observed in Asia and South Asia, and particularly in low- and middle-income regions. The burden of periodontitis increases with age, peaking in the 50–59 year age group, and it accounted for 6.2 million disability-adjusted life years (DALYs) globally in 2021 [3], underscoring its substantial public health impact. Beyond the health burden, periodontitis also exerts a major economic impact, with an estimated USD 54 billion in productivity losses annually and contributing significantly to the USD 442 billion global costs of oral diseases each year [4]. Clinically, the diagnosis of periodontitis still mainly relies on visual inspection, periodontal probing, and radiographic evaluation, which lack sufficient sensitivity and specificity for assessing current disease activity or predicting future progression. In terms of therapy, conventional approaches including nonsurgical treatment, surgical interventions, and adjunctive medications can control inflammation and slow disease progression, but they are prone to recurrence, offer limited long-term efficacy, and fail to achieve functional regeneration of lost tissues [5,6,7,8]. The primary feature of periodontitis is progressive destruction of periodontal supporting tissues. As the predominant cause of tooth loss in adults, periodontitis severely impairs mastication, facial aesthetics, and quality of life [9]. Importantly, increasing evidence demonstrates that periodontitis is closely associated with systemic diseases, including cardiometabolic diseases [10], neurodegenerative disorders [11,12], autoimmune diseases [13], and cancer [14,15], suggesting its broader clinical significance.

The pathogenesis of periodontitis is driven by complex interactions between polymicrobial communities and host immune responses [16]. These interactions are largely mediated by the secretome of both host and microbial cells, among which EVs have attracted considerable attention [17]. EVs are nanoscale lipid bilayer vesicles that carry nucleic acids, proteins, lipids, and other bioactive molecules [18,19], and they are increasingly recognized as critical mediators in the progression of periodontitis as well as potential contributors to associated systemic diseases [20,21]. Notably, EVs secreted from oral pathogens can encapsulate a variety of virulence factors and deliver them into host cells, thereby modulating immune responses and aggravating tissue destruction at local and distant sites [22,23]. Additionally, the double-membrane structure of EVs protects their cargo from enzymatic degradation [24], rendering EVs derived from saliva and GCF promising candidates for periodontal diagnostics [25]. Furthermore, MSC-EVs hold therapeutic potential in periodontitis due to their low immunogenicity, high safety, and multiple bioactivities [26]. Recently, engineering approaches have been developed to increase EV yield and enhance their therapeutic efficacy, enabling sustained, precise, and effective treatment in periodontitis [27,28].

We conducted a literature search primarily in PubMed for all publications related to EVs and periodontitis up to August 2025, with a particular focus on studies published in the last 5 years. The search was performed using combinations of keywords such as “extracellular vesicles”, “exosomes”, “periodontitis”, “gingival crevicular fluid”, “saliva”, “diagnostic biomarkers”, and “therapy”. We excluded non-English publications, inaccessible full texts, duplicates, meeting abstracts, and policy papers. Eligible studies were peer-reviewed original articles and reviews addressing the pathogenic, diagnostic, or therapeutic roles of EVs in periodontitis. In this review, we outline the roles of EVs in the pathogenesis of periodontitis and related systemic diseases, encompassing both BEVs and CEVs. We then discuss the diagnostic potential of EVs derived from saliva and GCF for periodontitis. We also summarize engineering strategies to optimize the therapeutic efficacy of MSC-EVs in periodontitis. Finally, we present the key challenges and future perspectives of EV-based applications in periodontitis.

## 2. Pathogenic Roles of BEVs in Periodontitis and Associated Systemic Diseases

BEVs are conventionally classified as outer membrane vesicles, outer–inner membrane vesicles, and cytoplasmic membrane vesicles, depending on their biogenetic pathways including outer membrane blebbing and explosive cytolysis [29]. Structurally, BEVs are spherical nanoscale vesicles with a double-layered membrane, enriched in pathogen-associated molecular patterns such as lipopolysaccharides (LPS) [30], toxins such as gingipains [31] and leukotoxin [32], nucleic acids [33], and other virulence factors, which enable them to trigger strong inflammatory responses and promote tissue destruction [34].

## 3. BEVs and Periodontitis

The gingival epithelium is a physical barrier serving as a critical frontline defense against microbial invasion. Gingival epithelial cells responded strongly to *Porphyromonas gingivalis* (*P. gingivalis*) EVs, which activated MAPK (Erk1/2, JNK, p38) and STING signaling pathways, resulting in elevated production of IL-6 and IL-8 to exacerbate epithelial inflammation and compromise barrier function [35]. EVs from *Filifactor alocis* (*F. alocis*) stimulated the release of cytokines including CXCL1, IL-6, IL-8, and GM-CSF in human oral keratinocytes, mimicking whole-bacterial stimulation [36]. Oral fibroblasts, another essential component of the connective barrier, internalized *P. gingivalis* EVs, which dose-dependently suppressed fibroblast proliferation and growth [37].

Beyond barrier disruption, BEVs profoundly influence innate and adaptive immune defenses, driving a proinflammatory state in periodontal tissues. Neutrophils, as the first line of defense, underwent degranulation and released antimicrobial components such as LL-37 and myeloperoxidase, which were subsequently degraded by *P. gingivalis* EVs-bound gingipains, thereby promoting bacterial survival [38]. EVs secreted by *Aggregatibacter actinomycetemcomitans* (*A. actinomycetemcomitans*) can induce the formation of neutrophil extracellular traps (NETs) [39]. Moreover, EVs shed from dental biofilms strongly induce neutrophil extracellular trap formation via a noncanonical cytosolic LPS/caspase-4/11/Gasdermin D pathway [40]. Following neutrophil activation, macrophages are another key target. Interestingly, *P. gingivalis* infection alone failed to trigger inflammasome activation in macrophages. In contrast, macrophages stimulated with *P. gingivalis*-EVs exhibited robust inflammasome activation, characterized by caspase-1 cleavage, abundant production of IL-1β and IL-18, and marked actate dehydrogenase release together with positive 7-AAD staining, indicative of pyroptotic cell death [41]. *A. actinomycetemcomitans*-EVs activated macrophages through the TLR-8/NF-κB signaling axis, leading to inflammasome activation and enhanced TNF-α release [42]. EVs derived from *Tannerella forsythia* (*T. forsythia*) activated macrophages through toll-like receptor 2 (TLR2) signaling and *T. forsythia* was also found to actively release EVs upon exposure to macrophage-derived soluble factors, suggesting a dynamic host–pathogen interaction that further amplifies periodontal inflammation [17]. EVs derived from *Fusobacterium nucleatum* (*F. nucleatum*) drove polarization toward the proinflammatory M1 phenotype. This M1-skewed response amplified local tissue destruction and accelerated alveolar bone loss [43]. *F. alocis* EVs markedly induced the expression of multiple inflammatory cytokines in macrophages, exhibiting immunostimulatory activity comparable to that of whole bacterial cells [36]. Further downstream, dendritic cells (DCs), as crucial antigen-presenting cells bridging innate and adaptive immunity, are highly susceptible to BEVs’ effects [44]. EVs from red complex pathogens (*P. gingivalis*, *T. forsythia*, *Treponema denticola*) promoted DC maturation by upregulating MHC class II and costimulatory molecules (CD80, CD86, CD40), and further skewed CD4^+^ T cell differentiation toward Th1 and Th17 lineages, thereby sustaining chronic inflammation and tissue degradation in periodontitis [45].

**Figure 1:**
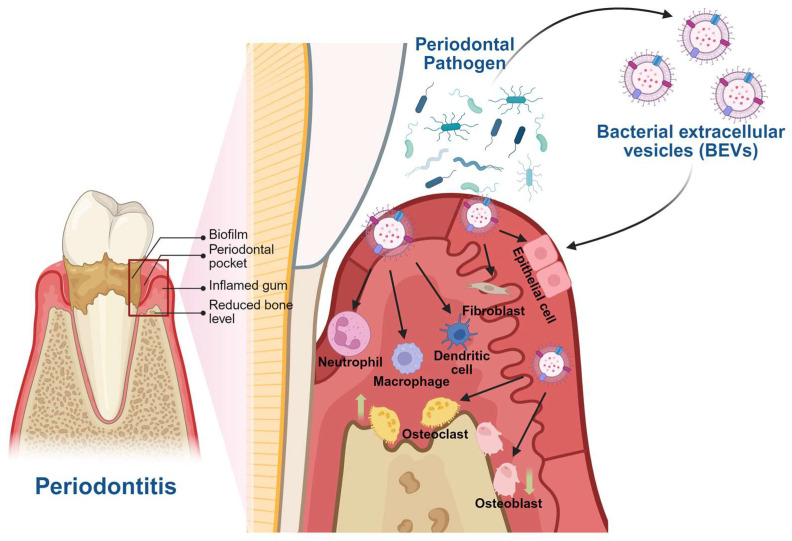
Pathogenic roles of BEVs in periodontitis. BEVs derived from periodontal pathogens penetrate epithelial and connective barriers, stimulating neutrophils, macrophages, and dendritic cells to induce excessive inflammatory responses, thereby sustaining chronic inflammation. Furthermore, BEVs perturb osteoimmune homeostasis by inhibiting osteogenesis and enhancing osteoclastogenesis, ultimately leading to progressive alveolar bone loss in periodontitis. Upward arrows represent upregulation, while downward arrows represent downregulation.

BEVs also perturb the osteoimmune balance, directly contributing to alveolar bone loss. On the osteogenic side, *F. alocis* EVs inhibited osteogenic differentiation of bone marrow mesenchymal stromal cells (BMSCs) via TLR2 signaling and increased the RANKL/OPG ratio, favoring osteoclastogenesis [46]. On the osteoclastic side, EVs from *P. gingivalis*, *T. forsythia*, and *Streptococcus oralis* promoted osteoclast differentiation through TLR2 activation by lipoproteins/LPS [47]. The BEVs derived from periodontal pathogens and their roles in periodontitis are summarized in Table 1 and Figure 1.

## 4. BEVs and Associated Systemic Diseases

BEVs have been implicated as key contributors to the pathogenesis and progression of Alzheimer’s disease. Gingivally exposed *P. gingivalis* EVs can translocate into the brain via the trigeminal nerve and periodontal bloodstream, contributing to cognitive decline [48]. *P. gingivalis* EVs delivered gingipains into brain microvascular endothelial cells, where they degraded tight junction proteins ZO-1 and occludin, thereby disrupting the blood–brain barrier (BBB) [49]. EVs from *A. actinomycetemcomitans*, administered by intragingival injection or EV-infused gel, have been shown to penetrate the BBB and elicit neuroinflammation. Their extracellular RNA cargo can activate TLR4 and TLR8 signaling pathways along with the downstream MyD88 pathway, inducing the production of proinflammatory cytokines including IL-6 and TNF-α [50]. Consistently, another in vitro study demonstrated that *A. actinomycetemcomitans* EV-associated RNAs activated the TLR8–NF-κB signaling axis in macrophages, leading to TNF-α expression [42]. Meanwhile, Hong et al. reported RNAs—but not DNA cargo—within *A. actinomycetemcomitans* EVs activated IL-6 and NF-κB signaling in microglia [51]. Notably, the glioblastoma-specific mutation EGFRvIII has been targeted in glioma models, where engineered BEVs carrying EGFRvIII epitopes suppressed tumor growth and elicited Th1-skewed immune responses characterized by CD4^+^ and CD8^+^ T-cell infiltration [52], underscoring the translational potential of BEV-based platforms in glioma immunotherapy.

Emerging evidence also highlights the detrimental impact of BEVs on vascular health. Gingipains presented on the surface of *P. gingivalis* EVs degraded endothelial cell–cell adhesins such as PECAM-1, increasing vascular permeability [53]. In diabetic retinopathy, *P. gingivalis* EVs accelerated blood-retinal barrier damage by inducing the expression of TNF-α, MMP-9, endothelial cell death, and barrier permeability via protease-activated receptor-2 [54].

Notably, BEVs also contribute to systemic bone loss. After intraperitoneal administration, *F. alocis* EVs accumulated in the long bones of mice and induced proinflammatory cytokine production, osteoclastogenesis, and bone resorption via TLR2 [55]. Moreover, *F. alocis* EVs inhibited the osteogenic differentiation of BMSCs by activating TLR2-mediated MAPK and NF-κB signaling, and further increased the RANKL/OPG ratio in BMSCs in a TLR2-dependent manner [46].

**Figure 2:**
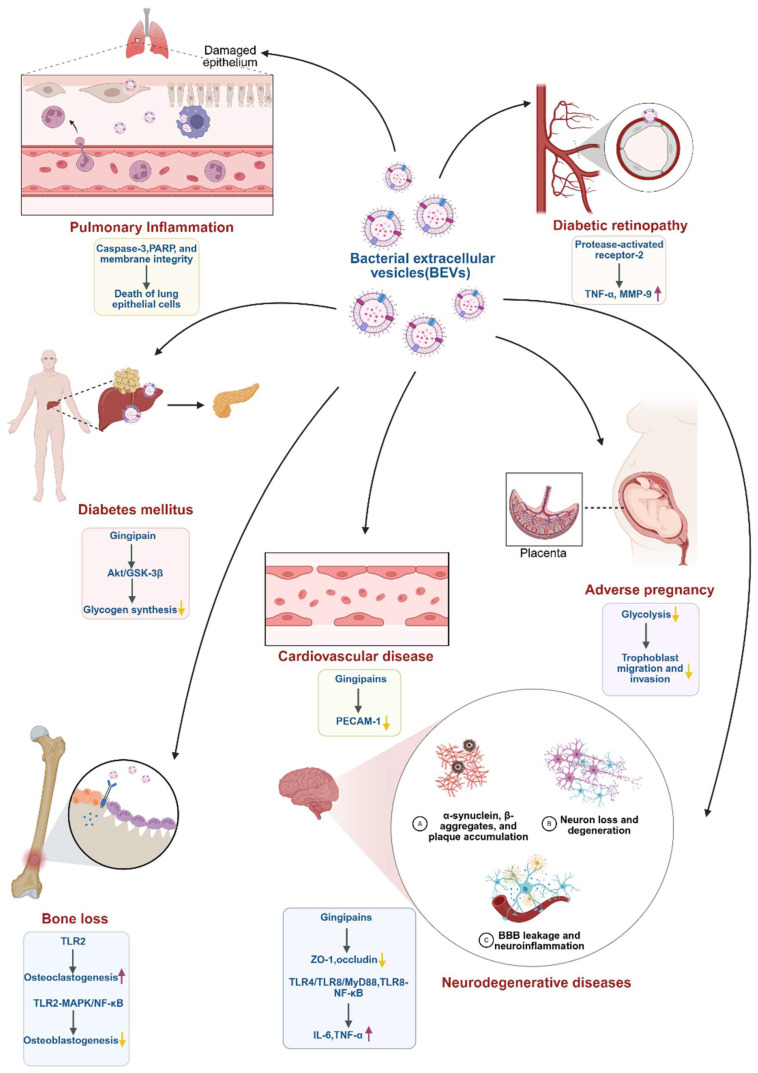
Promotional roles of BEVs in periodontitis related systemic diseases. BEVs extend their effects beyond the oral cavity, contributing to neurodegenerative disorders, cardiovascular disease, diabetes, diabetic retinopathy, pulmonary inflammation, systemic bone loss, and adverse pregnancy outcomes. BEVs provide a mechanistic link between periodontitis and multiple systemic conditions, highlighting their significance as both pathogenic mediators and potential therapeutic targets. Upward arrows represent upregulation, while downward arrows represent downregulation.

BEVs have been implicated in other diseases as well. For instance, *P. gingivalis* EVs induced cell death of lung epithelial cells via caspase-3 activation, PARP cleavage, and disruption of membrane integrity, indicating a link between periodontitis and aspiration pneumonia [56]. In the liver, *P. gingivalis* EVs impaired insulin-induced Akt/glycogen synthase kinase-3β (GSK-3β) signaling via gingipain-dependent pathways, leading to reduced glycogen synthesis and hyperglycemia, thereby contributing to diabetes development [57]. Moreover, *P. gingivalis* EVs are associated with adverse pregnancy outcomes. *P. gingivalis* EVs can be internalized by trophoblast cells, where they can suppress glycolysis and induce a metabolically quiescent state, impairing trophoblast migration and invasion [58]. The BEVs derived from periodontal pathogens and their roles in periodontitis-related systemic diseases are summarized in Table 2 and Figure 2.

## 5. Pathogenic Roles of CEVs in Periodontitis and Associated Systemic Diseases

**Figure 3:**
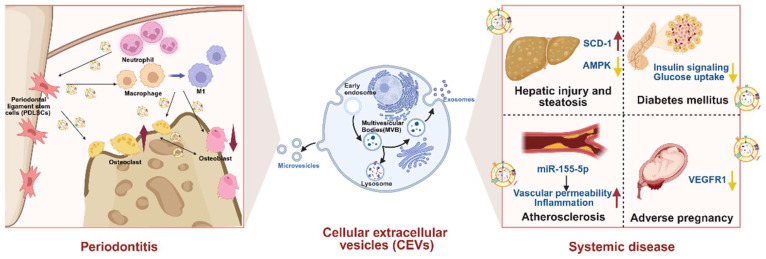
The roles of EVs derived from periodontal cells in the pathogenesis of periodontitis and related systemic diseases. CEVs modulate immune responses, impair osteogenesis, and promote osteoclastogenesis, thereby exacerbating periodontal inflammation and alveolar bone loss. Beyond local effects, CEVs influence systemic pathology by disrupting hepatic metabolism, impairing insulin signaling, increasing vascular permeability and inflammation, and inhibiting placental angiogenesis, linking periodontitis with hepatic injury, diabetes, atherosclerosis, and adverse pregnancy outcomes. Upward arrows represent upregulation, while downward arrows represent downregulation.

EVs are naturally secreted by virtually all cell types and are conventionally classified into endosome-derived exosomes, plasma membrane-derived microvesicles, and apoptotic bodies according to the biogenesis and release pathways [59,60]. EVs may undergo clathrin-dependent, caveolin-dependent, lipid raft–mediated endocytosis, macropinocytosis, and phagocytosis, as well as direct membrane fusion, thereby enabling their cargo to be released into the cytoplasm [61]. The cargo composition of EVs (such as proteins, transcription factors, nucleic acids, etc.) varies with the parental cell type, physiological conditions, and biogenesis [62,63,64]. Increasing evidence indicates that EVs derived from periodontal cells not only aggravate local periodontal damage (Table 3 and Figure 3) but also contribute to the progression of related systemic diseases (Table 4 and Figure 3).

## 6. CEVs and Periodontitis

During the progression of periodontitis, stem cell-derived EVs act as inflammatory amplifiers by aberrantly activating immune cells. In the inflamed microenvironment, EVs from Gli1^+^ MSCs can stimulate neutrophil activation via the CXCL1–CXCR2 axis, thereby exacerbating alveolar bone destruction [65]. LPS-stimulated periodontal ligament stem cells (PDLSCs) released EVs that drive macrophage polarization toward the proinflammatory M1 phenotype through multiple mechanisms [66]. For instance, miR-433-3p activated TLR2/TLR4/NF-κB signaling, while miR-143-3p inhibited PI3K/AKT and concomitantly activated NF-κB, collectively promoting M1 polarization, fibroblast apoptosis, and alveolar bone resorption [67,68].

In addition to modulating immune responses, EVs derived from various cell types impair osteogenesis and exacerbate bone loss [82]. Neutrophil-derived EVs inhibited the osteogenic differentiation of PDLSCs by delivering miR-223 and regulating the cGMP–PKG pathway [69]. EVs from inflammatory macrophages transferred damaged mitochondria to bone marrow mesenchymal stem cells (BMSCs), thereby impairing osteogenesis through the LCN2/OMA1/OPA1 axis [70]. Osteoclast-derived EVs enriched in miR-5134-5p suppressed JAK2/STAT3 signaling, leading to a marked reduction in osteogenic activity [71].

EVs also regulate osteoclast differentiation to modulate alveolar bone homeostasis. EVs from inflammatory PDLSCs enriched in RANKL and TNF-α strongly promoted osteoclastogenesis [72]. Osteoblast-derived EVs delivered the long noncoding RNA MALAT1, which modulated the miR-124/NFATc1 axis to enhance osteoclast differentiation [73]. *P. gingivalis* LPS-stimulated BMSCs secreted EVs that downregulated miR-151-3p and activated PAFAH1B1 signaling, further facilitating osteoclastogenesis [74]. In contrast, M2 macrophage-derived EVs carrying miR-1227-5p suppressed osteoclast-associated receptor (OSCAR) expression and inhibited osteoclast formation [75].

## 7. CEVs and Associated Systemic Diseases

EVs released from *P. gingivalis* LPS-stimulated periodontal cells, including macrophages and periodontal ligament fibroblasts, activated stearoyl-CoA desaturase-1 (SCD-1) and suppressed AMPK signaling in hepatocytes, thereby inducing hepatic injury and steatosis [76]. Gingival extracellular vesicles generated by polymicrobial periodontal infection disrupted adipose tissue function, as evidenced by reduced AKT phosphorylation, decreased adiponectin and leptin levels, and downregulation of genes related to adipogenesis and lipogenesis [77]. Plasma EVs from patients with periodontitis impaired insulin signaling and glucose uptake in hepatoma cells, exacerbating insulin resistance in type 2 diabetes [78,79]. Furthermore, plasma EVs enriched in miR-155-5p may increase vascular permeability and inflammation, accelerating carotid atherosclerosis [80]. In addition, EVs released by *P. gingivalis* infected macrophages have been shown to disrupt placental angiogenesis and downregulate VEGFR1 expression, leading to adverse pregnancy outcomes [81]. Collectively, these findings reinforce the pathological link between periodontitis and systemic diseases, and highlight periodontal cell-derived EVs as promising therapeutic targets. Current evidence indicates that in periodontitis, pathological CEVs are predominantly released in response to polymicrobial infection and bacterial components such as LPS, thereby contributing to systemic disease progression. While the possibility that BEVs directly regulate CEVs release is intriguing, current evidence is lacking, and future studies are needed to elucidate this potential interaction.

## 8. Diagnostic Role of EVs in Saliva and GCF

**Figure 4:**
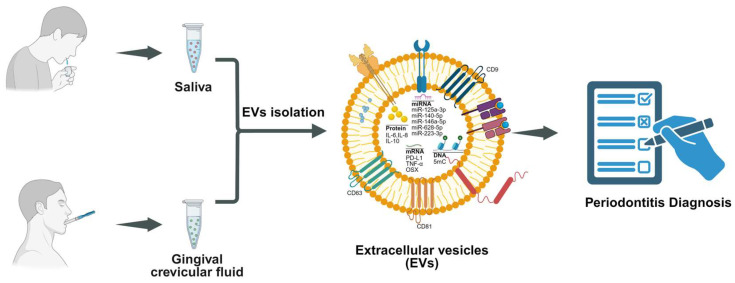
EVs derived from saliva and GCF as diagnostic biomarkers for periodontitis. GCF-EVs sensitively reflect local inflammation and tissue destruction, while salivary EVs capture both site-specific and systemic changes. Their molecular cargos, including microRNAs, mRNAs, proteins, and surface markers, show significant alterations in periodontitis, highlighting their utility for early detection, staging, and risk assessment.

Currently, the diagnosis of periodontitis mainly depends on clinical parameters such as plaque index, gingival index, bleeding on probing, periodontal pocket depth, radiographic alveolar bone loss, and tooth mobility [9,83,84]. However, these assessments largely provide historical data on periodontal tissue rather than predicting future disease activity, thereby limiting early detection and timely intervention. The molecular content of EVs in body fluids varies depending on whether cells are in a physiological or pathological state [85,86]. GCF is in direct contact with periodontal lesions and can sensitively reflect local inflammatory and tissue-destructive changes, while saliva, as a composite oral fluid, reflects both site-specific and overall oral health status. Importantly, both fluids can be collected non-invasively and painlessly [87,88,89]. EVs derived from GCF and saliva not only mirror the periodontal microenvironment but also may capture systemic pathological alterations, highlighting their potential as powerful tools for early disease screening, staging, and risk assessment (Figure 4) [90,91].

## 9. EVs in GCF

Compared with healthy and gingivitis subjects, the total concentration of EVs in GCF was significantly increased in periodontitis, whereas no significant difference was observed in saliva-derived EVs. The exosomal marker CD63 was also elevated in the GCF of periodontitis patients [92]. Previous studies have also demonstrated that GCF-EVs contain host-derived and bacterial-derived components, including cytokines, proteases, and nucleic acids, which are closely associated with periodontal inflammation and tissue destruction [93,94]. In addition, GCF-EVs were increased in both gestational diabetes mellitus (GDM) and periodontitis, and their proteomic cargos included peptides of both host and bacterial origin that participated in immune–inflammatory responses, glucose metabolism, and insulin signaling [95]. Collectively, these findings highlight the potential of GCF-EVs as biomarkers for the early diagnosis and monitoring of periodontitis and its related systemic diseases.

## 10. EVs in Saliva

Significant alterations in salivary EV composition have been observed in periodontitis and after initial periodontal therapy, involving microRNAs (miRNAs), messenger RNAs (mRNAs), proteins, and surface markers, all demonstrating considerable diagnostic potential [96]. At the miRNA level, 33 plasma miRNAs were significantly downregulated, while 1995 salivary miRNAs (1985 downregulated and 10 upregulated) were differentially expressed in periodontitis. Among them, miR-let-7d, miR-126-3p, and miR-199a-3p in plasma, and miR-125a-3p in saliva (all AUC = 1) in a pilot study of only 16 participants (8 periodontitis vs. 8 healthy), although the authors noted that these findings require validation in larger cohorts [97]. Compared with miRNAs from whole saliva, salivary EVs-derived miR-140-5p, miR-146a-5p, and miR-628-5p were markedly upregulated in periodontitis patients, each showing strong diagnostic power (AUC > 0.9) [98]. Xie et al. reported decreased miR-223-3p in salivary EVs and proposed its role in regulating GSDMD-mediated pyroptosis via NLRP3, suggesting potential for periodontitis diagnosis [99]. At the mRNA level, salivary exosomal PD-L1 was elevated and varied across the stages of periodontitis, indicating potential for both diagnosis and staging [100]. Meanwhile, osterix (OSX) mRNA decreased and TNF-α mRNA increased in periodontitis, showing moderate discriminatory capacity (AUC > 0.72) [101]. At the protein level, proteomic analyses revealed that proteins isolated from the saliva EVs of young adults with severe periodontitis were enriched in pathways related to innate immunity, cytolysis, and complement activation [102]. Han et al. found that EVs from periodontitis patients contained higher IL-6 and IL-8, and lower IL-10, reflecting a proinflammatory state in periodontitis [103]. Moreover, global 5mC hypermethylation was elevated in periodontitis, demonstrating high diagnostic accuracy (AUC = 1) in a pilot study of 22 participants, though age and smoking status differed among groups and no external validation was performed [104]. Regarding surface markers, significantly decreased CD9 and CD81 were detected in periodontitis and correlated with disease stage, although another study reported an increase in the CD9^+^ subpopulation [101], suggesting that the diagnostic value of CD9 requires further validation [105].

## 11. Strategies to Improve Therapeutic Function of MSC-EVs in Periodontitis

**Figure 5:**
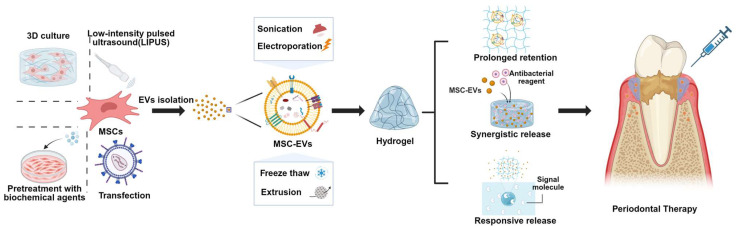
Main strategies for enhancing periodontal tissue repair with MSC-EVs, paving the way for their clinical application in periodontitis. Pretreatment, genetic modification, and microenvironmental modulation of parental cells improve EV yield and bioactivity, while sonication, electroporation, freeze–thaw, and extrusion facilitate therapeutic cargo loading. Incorporation of MSC-EVs into hydrogels enables prolonged retention, synergistic release with antibacterial agents, and stimulus-responsive delivery, thereby improving therapeutic efficiency and promoting periodontal regeneration.

Many studies have demonstrated that MSC-EVs promote the repair of complex periodontal tissues through diverse bioactivities, including enhanced cell proliferation [106,107], migration [108], angiogenesis [109], and multilineage differentiation [110]. These underlying mechanisms have been comprehensively summarized in several reviews [111,112,113]. Nevertheless, the clinical translation of EVs remains hampered by challenges such as low production efficiency, rapid clearance, limited tissue specificity, and unpredictable cargo loading [27,114,115]. To address these limitations, recent studies have investigated various engineering strategies to enhance the therapeutic efficacy of EVs (Figure 5) [116].

## 12. Pretreatment Approaches of Parental Cells

The yield and functional cargo of natural MSC-EVs are inherently limited. Pretreatment with biochemical agents significantly enhances the therapeutic potential of MSC-EVs. For instance, metformin increased PDLSC-EVs secretion and improved their osteogenic and anti-inflammatory activities [117]. Psoralen upregulated miR-125b-5p in PDLSC-EVs, thereby promoting osteogenic differentiation [118]. Gallic acid alleviated inflammation in PDLSCs and enhanced the pro-osteogenic effects of their EVs [119]. EVs from LPS-preconditioned dental follicle cells attenuated alveolar bone loss by modulating RANKL/OPG-mediated osteoclastogenesis and inducing M2 macrophage polarization, while also protecting periodontal ligament cells (PDLCs) against apoptosis and dysfunction through JNK and p38 signaling pathways [120,121].

Genetic modification of parental cells represents another effective strategy to improve EV bioactivity. EVs from FoxO1-overexpressing PDLSCs were enriched in FoxO1 and demonstrated enhanced osteogenic capacity while suppressing inflammation [122]. CXCR4-transfected cells generated EVs with elevated CXCR4 expression, endowing them with improved “homing” ability toward inflammatory sites [123]. Chen et al. reported that EVs derived from P2X7R-modified PDLSCs restored the function of inflammation-compromised PDLSCs by transferring miR-3679-5p, miR-6515-5p, and miR-6747-5p [124]. Additionally, the cationic antimicrobial peptide DP7-C has been applied as a transfection carrier to deliver miR-26a and miR-21b into parental cells, producing EVs enriched with these therapeutic miRNAs [125,126].

Parental cells can also be modulated through the culture microenvironment to optimize EV production. For example, dental pulp stem cells cultured in three-dimensional systems secreted EVs that restored the Th17/Treg balance in inflamed periodontal tissues via the miR-1246/Nfat5 axis [127]. Moreover, low-intensity pulsed ultrasound (LIPUS) stimulation of stem cells from apical papilla promoted the secretion of miR-935-enriched EVs with enhanced osteogenic and anti-inflammatory properties [128].

## 13. Direct Engineering Approaches of EVs

Recent studies have increasingly explored direct engineering strategies to load therapeutic agents into EVs for the treatment of periodontitis. Approaches such as sonication, electroporation, freeze–thaw treatments, and extrusion have been employed to enhance EV drug-loading efficiency [129,130,131]. Notably, melatonin-loaded M2 macrophage-derived EVs restored osteogenic and cementogenic differentiation of inflamed PDLCs by alleviating excessive endoplasmic reticulum stress and unfolded protein response [132]. Aspirin-loaded PDLSC-derived EVs improved the periodontal immune microenvironment by promoting oxidative phosphorylation (OXPHOS) in macrophages, thereby reducing alveolar bone loss [133]. Likewise, methotrexate-loaded EVs derived from human umbilical cord mesenchymal stem cells suppressed proinflammatory macrophage activation through acyl-CoA synthetase-1 (ACSL1) downregulation and OXPHOS restoration, ultimately improving therapeutic outcomes in periodontitis [134,135].

## 14. MSC-EVs Combined with Biomaterials Promote Periodontal Tissue Regeneration

The limited half-life of EVs following administration, coupled with their rapid diffusion from the site of delivery, compromises therapeutic benefits. To overcome this, hydrogels with good biocompatibility and porous architectures have been developed as promising delivery systems for EVs. Among them, Hydrogels composed of chitosan (CS) and β-sodium glycerophosphate (β-GP) are widely recognized for their injectability and ability to undergo thermosensitive gelation at physiological temperature, making them suitable for repairing irregular tissue defects. Incorporation of dental pulp stem cell-derived EVs into CS hydrogels accelerated alveolar bone and periodontal epithelial regeneration in murine models of periodontitis [136]. Sun et al. further improved CS hydrogels by adding gelatin, which shortened gelation time, while erythropoietin-stimulated EVs encapsulated in CS/β-GP/gelatin hydrogels ensured sustained release and prolonged retention in vivo [137]. To address the limited antimicrobial activity of EVs, Su et al. incorporated copper ions (Cu^2+^) and EVs from exfoliated deciduous teeth into hyaluronic acid hydrogels, achieving synergistic antibacterial and osteogenic effects [138]. Similarly, Xie et al. developed thermosensitive hydrogels co-loaded with BMSC-derived EVs and calcium peroxide nanoparticles, which provided sustained oxygen release, thereby alleviating anaerobic infections and promoting periodontal regeneration [139]. In addition, controlled-release systems have been designed for pathological site-specific delivery. For example, EVs derived from gingival mesenchymal stem cells immobilized on microparticles via matrix metalloproteinase (MMP)-sensitive linkers enabled targeted release at inflammatory sites, resulting in localized immunomodulation and significantly improved periodontal regeneration [140].

## 15. Summary and Prospect

Oral BEVs play pivotal roles in the initiation and progression of periodontitis and are increasingly recognized as contributors to related systemic diseases. Accumulating evidence indicates that BEVs can degrade connective tissue, amplify inflammatory responses, and disturb alveolar bone homeostasis, thereby exacerbating periodontal destruction. Despite these advances, the precise molecular mechanisms underlying BEV-mediated pathogenicity remain insufficiently understood and require further in-depth investigation. To enhance reproducibility and comparability across studies, it is crucial to optimize BEV isolation and purification techniques and to establish standardized criteria for their characterization. Furthermore, the application of multi-omics strategies will be instrumental in defining the detailed cargo composition and functional heterogeneity of BEVs derived from diverse bacterial species. Such approaches may ultimately facilitate the identification of specific pathogenic signatures and promote the development of innovative anti-BEV therapeutic strategies for periodontitis and related systemic diseases.

EVs derived from saliva and GCF hold great promise as non-invasive diagnostic biomarkers for periodontitis. However, several important challenges remain to be addressed before clinical translation. Methodological standardization is critical to improve reproducibility. According to the Minimal Information for Studies of Extracellular Vesicles (MISEV) guidelines from the International Society for Extracellular Vesicles (ISEV), oral EV studies should follow standardized workflows covering pre-analytical variables (sample source, collection method, storage), isolation strategies (e.g., differential ultracentrifugation, density gradients, size-exclusion chromatography, fluid flow-based separation, charge and molecular recognition-based separations), and comprehensive characterization, including particle analysis, morphology, and molecular markers. To ensure rigor, both positive markers (CD9, CD63, CD81, TSG101, ALIX, syntenin-1) and negative markers (calnexin, GM130, GRP94) should be evaluated to confirm vesicular identity and exclude contaminants. Furthermore, oral biofluids present notable pitfalls [141,142,143,144]. Oral biofluids is prone to contamination by food debris and oral bacteria, and contains endogenous enzymes such as amylase. These factors can obscure biomarker detection and reduce sensitivity and specificity by promoting rapid protein/RNA degradation and denaturation [145,146].

EVs in saliva and GCF originate from multiple host cell types and diverse bacterial species, generating substantial heterogeneity that complicates interpretation. Advanced fractionation and isolation strategies are urgently needed to accurately separate bacterial EVs from host-derived vesicles and to resolve distinct EV subpopulations. The use of immunoaffinity capture with BEV-specific markers such as LPS and lipoteichoic acid (LTA) and CEV-specific markers such as CD9, CD63, CD81, TSG101, and ALIX represents a promising approach [141,147]. In addition, emerging single-EV multi-omics technologies may uncover source-specific molecular signatures and define robust biomarker panels with high diagnostic accuracy. Importantly, the cargo of oral EVs reflects both the periodontal pathogen burden and the host inflammatory status. Therefore, integrating EV-based analyses with conventional microbiological and inflammatory biomarkers may enable a more comprehensive assessment of periodontitis activity. Finally, large-scale, multicenter clinical studies across different age groups, genders, and ethnic backgrounds are required to validate findings and establish standardized diagnostic thresholds, thereby enabling early periodontitis screening and personalized risk assessment.

MSC-EVs have emerged as a compelling alternative to conventional stem cell therapy and are increasingly recognized as a promising therapeutic modality for periodontal regeneration. The yield and functional activity of MSC-EVs are strongly influenced by parental cell type, culture conditions, and passage number [148,149]. Furthermore, large-scale production and purification of MSC-EVs remain costly. Advances in bioreactor technologies and standardized isolation protocols may improve production efficiency and cost-effectiveness [150,151]. For GMP-grade production, closed and scalable bioreactor systems with full process validation are required, and batch release testing must confirm identity, purity, sterility, potency, and safety. Storage and stability also represent critical challenges, as EVs are stable at −80 °C but prone to aggregation or degradation at higher temperatures, underscoring the need for standardized release criteria and storage conditions to ensure reproducibility [152,153].

In terms of safety, preclinical studies indicate that MSC-EVs are generally low-immunogenic. However, potential risks including off-target effects, unpredictable biodistribution, and excessive immunosuppression must be carefully addressed. Therefore, rigorous safety evaluations and long-term immunological assessments should be carefully evaluated and monitored. Systematic investigations of in vivo pharmacokinetics and pharmacodynamics are essential to clarify biodistribution, clearance profiles, and optimal dosing regimens. Considering the complexity of periodontal tissue repair, future strategies should also emphasize multifunctional and engineered approaches that integrate osteogenic, angiogenic, immunomodulatory, and antibacterial properties, possibly through the development of EV-based combinatorial biomaterials to achieve safer and more predictable therapeutic outcomes.

Translation into clinical practice will require carefully designed first-in-human trials. One possible design is a randomized, double-blind, placebo-controlled study in systemically healthy adults with periodontitis. Exclusion criteria would include smoking, diabetes, pregnancy, corticosteroid or contraceptive use, and poor oral hygiene. Patients who had completed initial periodontal therapy, such as scaling and root planing, could then be randomized to receive either local MSC-EV administration or placebo. Therapeutic efficacy would be assessed by monitoring changes in gingival inflammation, probing pocket depth, clinical attachment level, and alveolar bone level.

Collectively, EVs are pivotal in the pathogenesis, diagnosis, and therapy of periodontitis. Nonetheless, several key barriers currently limit their clinical application (Table 5). Continued efforts are essential to accelerate the clinical translation of EV-based diagnostics and therapeutics for periodontitis.

## Figures and Tables

**Table 1:** BEVs derived from periodontal pathogens and their roles in periodontitis.

BEVs and Periodontitis							
EV Source	Cargo	Target Cell	Pathway	Readouts	Model	Strength of Evidence	Ref.
*P. gingivalis*	Gingipains	Gingival epithelial cells	MAPK(Erk1/2, JNK, p38) and STING	↑IL-6 and ↑IL-8	In vitro gingival epithelial cells assay	In vitro	[35]
*F. alocis*	Lipoproteins, FACIN, and autolysins	Monocytes and oral keratinocyte cells	/	↑CCL1, ↑CCL2, ↑MIP-1, ↑CCL5, ↑CXCL1, ↑CXCL10, ↑ICAM-1, ↑IL-1β, ↑IL-1ra, ↑IL-6, ↑IL-8, ↑MIF, ↑SerpinE, and ↑TNF-α in human monocytes and ↑CXCL1, ↑G-CSF, ↑GM-CSF, ↑IL-6, and ↑IL-8 in human oral keratinocyte cells	In vitro monocytes and oral keratinocyte cells assay	In vitro	[36]
*P. gingivalis*	Gingipains	Oral fibroblasts	/	The internalization of Porphyromonas gingivalis bEVs by oral fibroblasts ↓Fibroblast proliferation and growth	In vitro oral gingival fibroblasts assay	In vitro	[37]
*P. gingivalis*	Gingipains	Neutrophils	/	↑LL-37, ↑MPO	In vitro neutrophils assay	In vitro	[38]
*A. actinomycetemcomitans*	/	Neutrophils	/	The formation of neutrophil extracellular traps (NETs)	In vitro neutrophils assay	In vitro	[39]
Dental biofilm	LPS	Neutrophils	LPS/caspase-4/11/Gasdermin D	The formation of neutrophil extracellular traps (NETs)	In vitro neutrophils assay Human periodontal tissues, blood, and biofilm samples	In vitro→Human	[40]
*P. gingivalis*	/	Macrophages	Caspase-1	↑TNFα, ↑IL-12p70, ↑IL-6, ↑IL-10, ↑IFNβ, ↑nitric oxide, ↑IL-1β, and ↑IL-18 The activation of the inflammasome and pyroptotic cell death pathways	In vitro macrophages assay	In vitro	[41]
*T. forsythia*	BspA, sialidase, GroEL, and various bacterial lipoproteins	Macrophages	TLR2	↑Pro-inflammatory cytokines and ↑inflammatory mediators	In vitro macrophages assay	In vitro	[17]
*F. nucleatum*	/	Macrophages and gingival fibroblasts	/	Polarization of macrophages toward the proinflammatory M1 phenotype ↑TNF-α,↑iNOS, and ↑LDH	In vitro macrophages and gingival fibroblasts assay In vivo mouse periodontitis model	In vitro→Animal	[43]
Red complex pathogens	/	Bone marrow-derived dendritic cells (BMDCs)	/	↑MHC class II, ↑CD80, ↑CD86, ↑CD40, ↑IL-1, ↑IL-6, ↑IL-23, and ↑IL-12p70 Maturation of BMDCs	In vitro BMDCs assay	In vitro	[45]
*F. alocis*		Bone-derived mesenchymal stromal cells (BMSCs)	TLR2	↑RANKL/OPG	In vitro BMSCs assay	In vitro	[46]
Periodontal pathogens and oral commensal	Lipoproteins and LPS	Osteoclasts	TLR2	↑Expression of osteoclastogenic cytokines ↑Osteoclast differentiation	In vitro osteoclast precursors assay	In vitro	[47]

Note: ↑ indicates upregulation; ↓ indicates downregulation.

**Table 2:** BEVs derived from periodontal pathogens and their roles in periodontitis-related systemic diseases.

BEVs and Associated Systemic Diseases								
Disease	EV Source	Cargo	Target Cell	Pathway	Readouts	Model	Strength of Evidence	Ref.
Neurodegenerative diseases	*P. gingivalis*	Neurotoxic GPs, inflammation-inducible fimbria protein and LPS	BV2, SH-SY5Y, and peritoneal macrophages	/	↑TNF-α expression in the periodontal and hippocampus tissues ↑Hippocampal GP^+^Iba1^+^, ↑LPS^+^Iba1^+^, and ↑NF-κB^+^Iba1^+^ cell numbers ↓BDNF, ↓claudin-5, ↓N-methyl-D-aspartate receptor expression, and ↓BDNF^+^NeuN^+^ cell number ↑Blood LPS and TNF-α	In vitro BV2, SH-SY5Y, and peritoneal macrophages assay In vivo mouse models of cognitive impairment induced by *P.g* or pEV	In vitro→Animal	[48]
		Gingipains	hCMEC/D3	PAR2	↓ZO-1 and ↓occludin ↑Permeability of hCMEC/D3 cell monolayer	In vitro hCMEC/D3 assay	In vitro	[49]
	*A. actinomycetemcomitans*	RNA	Trigeminal ganglion (TG) neurons	TLR4, TLR8, MyD88	↑IL-6 and ↑TNF-α	In vivo mouse periodontitis model	Animal	[50]
		exRNA	Macrophages	TLR8, NF-κB	↑TNF-α	In vitro macrophages assay In vivo mouse heart injection OMV model	In vitro→Animal	[42]
			Microglia	NF-κB	↑IL-6	In vitro BV2 assay In vivo imaging model after OMV injection in mice	In vitro→Animal	[51]
Cardiovascular disease	*P. gingivalis*	Gingipains	Human microvascular endothelial cells (HMEC-1)		↓PECAM-1 ↑Vascular permeability	In vitro HMEC-1 cells assay In vivo zebrafish OMV injection model	In vitro→Animal	[53]
Diabetic retinopathy	*P. gingivalis*	/	Human retinal microvascular endothelial cells (HRMECs)	PAR-2	↑HRMECs inflammatory factors and ↑reactive oxygen species production Mitochondrial dysfunction, apoptosis, and altered endothelial permeability	In vitro HRMECs assay In vivo mouse diabetes model	In vitro→Animal	[54]
Bone loss	*F. alocis*	/	Committed osteoclast precursors (COCs)	TLR2	↑Proinflammatory cytokines, ↑Osteoclastogenesis, and ↑bone resorption	In vivo mice intraperitoneal injection of OMVs model	Animal	[55]
		/	Bone-derived mesenchymal stromal cells (BMSCs)	MAPK, NF-κB	↑RANKL/OPG	In vitro BMSCs assay	In vitro	[46]
Pneumonia	*P. gingivalis*	/	Lung epithelial cells	/	Caspase-3 activation and PARP cleavage ↓Tight junction proteins	In vitro lung epithelial cells	In vitro	[56]
Diabetes	*P. gingivalis*	Gingipains	HepG2	Insulin-induced Akt/glycogen synthase kinase-3β (GSK-3β)	↓Insulin sensitivity	In vitro HepG2 assay In vivo mouse intraperitoneal injection of OMVs model	In vitro→Animal	[57]
Adverse pregnancy	*P. gingivalis*	/	Trophoblast cells	/	↓Glycolytic pathways in the placenta, ↓placental, ↓fetal weight and ↓GLUT1	In vitro the first trimester trophoblast cells assay In vivo mouse pregnancy model	In vitro→Animal	[58]

Note: ↑ indicates upregulation; ↓ indicates downregulation.

**Table 3:** Roles of CEVs in the pathogenesis of periodontitis.

CEVs and Periodontitis							
EV Source	Cargo	Target Cell	Pathway	Readouts	Model	Strength of Evidence	Ref.
Gli1^+^ MSCs	/	Neutrophils	CXCL1–CXCR2 axis and NF-κB	↑ROS Aberrant activation of neutrophils	In vitro neutrophils assay In vivo mouse periodontitis model	In vitro→Animal	[65]
Periodontal ligament stem cells (PDLSCs)	/	Macrophages and periodontal ligament fibroblasts (PLFs)	/	Polarization of macrophages toward the proinflammatory M1 phenotype NLRP3 inflammasome activation	In vitro macrophages and PLFs assay In vivo mouse periodontitis model	In vitro→Animal	[66]
	miR-143-3p	Macrophages	PI3K/AKT/NF-κB	Polarization of macrophages toward the proinflammatory M1 phenotype	In vitro macrophages assay In vivo mouse periodontitis model	In vitro→Animal	[67]
	microRNA-433-3p	Macrophages	TLR2/TLR4/NF-κB p65	Polarization of macrophages toward the proinflammatory M1 phenotype	In vitro macrophages assay Human periodontal ligament stem cells isolation	In vitro→Human	[68]
Neutrophils	miR-223	Periodontal ligament stem cells (PDLSCs)	cGMP-PKG	Inhibit osteogenic differentiation of PDLSCs	In vitro PDLSCs assay	In vitro	[69]
Macrophages	mitochondria	Bone marrow mesenchymal stem cells (BMSCs)	LCN2/OMA1/OPA1	↑LCN2 Mitochondrial morphological changes in BMSCs Osteogenesis impairment in BMSCs	In vitro BMSCs assay In vivo mouse periodontitis model	In vitro→Animal	[70]
Osteoclasts	miR-5134-5p	Osteoblasts	JAK2/STAT3	↑miR-5134-5p ↓Runx2, ↓p-JAK2, and ↓p-STAT3 ↑Inflammatory factors mRNA expression ↓BV/TV ↑Cementoenamel junction and alveolar bone crest distance Morphological disruption of periodontal tissue Inflammatory cell infiltration	In vitro osteoblasts assay In vivo mouse periodontitis model	In vitro→Animal	[71]
Periodontal ligament stem cells (PDLSCs)	RANKL, TNF-α	Macrophages	NF-κB	↑Osteoclast differentiation	In vitro macrophages assay	In vitro	[72]
Osteoblasts	lnc-MALAT1	Macrophages	miR-124/NFATc1	The acceleration of the progression of osteoclastogenesis	In vitro macrophages assay	In vitro	[73]
Bone marrow mesenchymal stem cells (BMSCs)	miR-151-3p	Macrophages	miR-151-3p/PAFAH1B1	↑Osteoclastogenesis	In vitro macrophages assay	In vitro	[74]
M2-like macrophages	miR-1227-5p	Osteoclasts	/	↑miR-1227-5p ↓Osteoclast differentiation	In vitro Osteoclasts assay	In vitro	[75]

Note: ↑ indicates upregulation; ↓ indicates downregulation.

**Table 4:** Roles of CEVs in the pathogenesis of periodontitis-related systemic diseases.

CEVs and Associated Systemic Diseases								
Disease	EV Source	Cargo	Target Cell	Pathway	Readouts	Model	Strength of Evidence	Ref.
Hepatic steatosis	Macrophages and human periodontal ligament fibroblasts (hPDLFs)	/	HepG2	SCD-1/AMPK	↑SCD-1 and ↑Hepatocyte adipogenesis ↓AMPK	In vitro HepG2 assay In vivo rat periodontitis model	In vitro→Animal	[76]
Adipose tissue dysfunction	Gingival cells	/	Adipocytes	/	WAT dysfunction ↓Levels of AKT phosphorylation, ↓adiponectin, ↓leptin, and ↓genes associated with adipogenesis and lipogenesis	In vivo mouse oral and intraperitoneal injection models	Animal	[77]
Diabetes	Plasma	/	HepG2	Insulin signaling	↓p-AKT, ↓p-GSK3β, and ↓hepatic glycogen content	In vitro HepG2 assay In vivo rat diabetic model Human blood sample collection	In vitro→Animal→Human	[78]
	Osteocytes	miR-124-3p, Gal-3, and IL-6	Osteoblasts	/	The regulation of Gal-3 expression of osteoblasts	In vitro Osteoblasts assay In vivo rat diabetic model and periodontitis model Human saliva collection	In vitro→Animal→Human	[79]
Carotid atherosclerosis	Human umbilical vein endothelial cells (HUVECs)	miR-155-5p	Human aortic endothelial cells(HAECs)	/	↑Angiogenesis and ↑permeability of HAECs ↑Expression of angiogenesis, ↑permeability, and ↑inflammation genes The acceleration of the occurrence of carotid atherosclerosis	In vitro HAECs assay In vivo mouse intravenous injection of OMVs model Human tissue samples	In vitro→Animal→Human	[80]
Adverse pregnancy	Macrophages	/	Human umbilical vascular endothelial cells(HUVECs)	/	↓VEGFR1 Disoriented blood vessel alignment Impaired angiogenesis	In vitro HUVECs assay In vivo mouse intravenous injection of OMVs model	In vitro→Animal	[81]

Note: ↑ indicates upregulation; ↓ indicates downregulation.

**Table 5:** Summary of key advances and controversies in EV research on periodontitis.

Aspect	What’s New	What’s Controversial
BEVs	Systematic summary of BEVs promoting periodontitis via epithelial barrier disruption, immune-inflammatory amplification, osteogenesis inhibition, and osteoclastogenesis promotion, as well as their involvement in periodontitis-associated systemic diseases.	Mechanisms of BEVs crossing biological barriers, and their contribution to periodontitis and systemic diseases remain debated.
CEVs	Emphasis on periodontal cell derived EVs under inflammatory conditions, which promote periodontitis by aggravating inflammation and disturbing bone homeostasis, and further contribute to systemic disease progression.	Mechanisms of CEVs-mediated effects in periodontitis and related systemic diseases are still insufficiently defined.
EVs for Diagnosis	Updated summary of recent studies on GCF- and saliva-derived EVs as non-invasive biomarkers for detection, staging, and risk assessment.	The diagnostic biomarkers derived from GCF and saliva EVs for periodontitis have not yet been clearly identified.
EVs for Therapy	Highlight recent engineering strategies (pretreatment of parental cells, direct EV modification, biomaterial-based delivery) to improve EVs’ therapeutic function and achieve targeting and controlled release, thereby enhancing periodontal tissue regeneration.	Clinical translation of EVs is limited by low yield, rapid clearance, poor tissue specificity, and unpredictable cargo loading.

## Data Availability

No new data were created or analyzed in this study.

## Abbreviations

The following abbreviations are used in this manuscript:

EVs	extracellular vesicles
GCF	gingival crevicular fluid
MSC-EVs	mesenchymal stem cell–derived EVs
BEVs	bacterial extracellular vesicles
CEVs	cellular extracellular vesicles
LPS	lipopolysaccharides
*P. gingivalis*	*Porphyromonas gingivalis*
*T. forsythia*	*Tannerella forsythia*
*F. nucleatum*	*Fusobacterium nucleatum*
*F. alocis*	*Filifactor alocis*
*A. actinomycetemcomitans*	*Aggregatibacter actinomycetemcomitans*
DCs	dendritic cells
IL-6	interleukin-6
IL-8	interleukin-8
CXCL1	C-X-C motif chemokine ligand 1
GM-CSF	granulocyte–macrophage colony-stimulating factor
TLR2	toll-like receptor 2
MHC	major histocompatibility complex
BBB	blood–brain barrier
ZO-1	zonula occludens-1
TLR4	toll-like receptor 4
TLR8	toll-like receptor 8
TNF-α	tumor necrosis factor-alpha
PECAM-1	platelet endothelial cell adhesion molecule-1
MMP-9	matrix metalloproteinase-9
GSK-3β	glycogen synthase kinase-3β
CXCR2	C-X-C chemokine receptor type 2
PDLSCs	periodontal ligament stem cells
SCD-1	stearoyl-CoA desaturase-1
VEGFR1	vascular endothelial growth factor receptor 1
GDM	gestational diabetes mellitus
OSX	osterix
IL-10	interleukin-10
5mC	5-methylcytosine
PDLCs	periodontal ligament cells
FoxO1	forkhead box protein O1
CXCR4	C-X-C chemokine receptor type 4
OXPHOS	oxidative phosphorylation
ACSL1	acyl-CoA synthetase-1
CS	chitosan
β-GP	β-sodium glycerophosphate
MMP	matrix metalloproteinase
